# Effect of immunology biomarkers associated with hip fracture and fracture risk in older adults

**DOI:** 10.1186/s12979-023-00379-z

**Published:** 2023-10-18

**Authors:** Bernardo Abel Cedeno-Veloz, Lucía Lozano-Vicario, Fabricio Zambom-Ferraresi, Joaquín Fernández-Irigoyen, Enrique Santamaría, Alba Rodríguez-García, Roman Romero-Ortuno, Jaime Mondragon-Rubio, Javier Ruiz-Ruiz, Robinson Ramírez-Vélez, Mikel Izquierdo, Nicolás Martínez-Velilla

**Affiliations:** 1 Geriatric Department, Hospital Universitario de Navarra (HUN), 2 Navarrabiomed, Pamplona, Navarra, IdiSNA, 31008 Spain; 2 Navarrabiomed, Navarra Medical Research Institute, Pamplona, Navarra, 31008 Spain; 3 Department of Health Sciences, Public University of Navarra, Pamplona, Navarra, 31008 Spain; 4 CIBER of Frailty and Healthy Aging (CIBERFES), Instituto de Salud Carlos III, Madrid, 28029 Spain; 5 Clinical Neuroproteomics Unit, Navarrabiomed, Pamplona, 31008 Spain; 6 Discipline of Medical Gerontology, School of Medicine, Trinity College Dublin, Dublin, Ireland; 7 Department of Orthopaedics Clinics and Traumatology, University Hospital of Navarre (HUN), Pamplona, Navarra, 31008 Spain

**Keywords:** Cytokines, Hip fractures, Biomarkers, Prognosis, FRAX

## Abstract

Osteoporosis is a skeletal disease that can increase the risk of fractures, leading to adverse health and socioeconomic consequences. However, current clinical methods have limitations in accurately estimating fracture risk, particularly in older adults. Thus, new technologies are necessary to improve the accuracy of fracture risk estimation. In this observational study, we aimed to explore the association between serum cytokines and hip fracture status in older adults, and their associations with fracture risk using the FRAX reference tool. We investigated the use of a proximity extension assay (PEA) with Olink. We compared the characteristics of the population, functional status and detailed body composition (determined using densitometry) between groups. We enrolled 40 participants, including 20 with hip fracture and 20 without fracture, and studied 46 cytokines in their serum. After conducting a score plot and two unpaired t-tests using the *Benjamini-Hochberg* method, we found that Interleukin 6 (IL-6), Lymphotoxin-alpha (LT-α), Fms-related tyrosine kinase 3 ligand (FLT3LG), Colony stimulating factor 1 (CSF1), and Chemokine (C-C motif) ligand 7 (CCL7) were significantly different between fracture and non-fracture patients (p < 0.05). IL-6 had a moderate correlation with FRAX (*R*^2^ = 0.409, p < 0.001), while CSF1 and CCL7 had weak correlations with FRAX. LT-α and FLT3LG exhibited a negative correlation with the risk of fracture. Our results suggest that targeted proteomic tools have the capability to identify differentially regulated proteins and may serve as potential markers for estimating fracture risk. However, longitudinal studies will be necessary to validate these results and determine the temporal patterns of changes in cytokine profiles.

**Supplementary Information:**

The online version contains supplementary material available at 10.1186/s12979-023-00379-z.

## Introduction

Osteoporosis (OP) is delineated systemic skeletal disorder associated with a reduced quantity of bone mineral mass and the microarchitectural degradation of the bone’s tissue structure, which increases the risk of fragility fracture [1]. Due to its chronic nature and prevalence in an ageing population, OP has significant human and socioeconomic consequences, including morbi-mortality and disability [2]. Therefore, identifying high risk populations and exploring potential biomarkers associated related to bone changes is crucial for effective health promotion [3].

Clinical guidelines serve as a foundation for assessing fracture risk [1] and promoting early interventions. Nonetheless, the most frequently examined parameters, such as bone mineral density (BMD), bone turnover markers (BMT) and FRAX® [4], exhibit limited efficacy, particularly in older population. BMD has been extensively researched and is recognized as a conventional risk determinant for fractures, but its low sensitivity is one of the reasons why population-based screening for BMD is not recommended for risk fracture assessment [1]. Another contributing factor is the relatively weak correlation between the loss of BMD and the capability to accurately forecast the risk of fractures [5]. BTM does not enhance fracture risk or bone loss prediction within an individual and is primarily useful in monitoring oral bisphosphonate therapy [6] or other osteoporosis treatments. FRAX, despite its widespread usage as a simple and primary care-applicable tool for estimating fracture risk and first-choice tool in most of clinical guidelines [1], possesses a limitation in that it does not accommodate dose-response considerations for diverse risk factors [7, 8], potentially underestimating fracture risk [9], and is unsuitable for adults aged over 90 [4]. While FRAX advances fracture prognostication beyond the capabilities of Bone Mineral Density (BMD) measurements alone, the accuracy of its fracture risk prediction displays variation across distinct study populations [10]. Consequently, there is a compelling need to investigate innovative approaches for estimating fracture risk. Presently, a revised version of FRAX is under development, with the intention of addressing the aforementioned limitations [11].

Bone loss in the ageing population is commonly attributed to its endocrine origin. However, comorbidities, genetics, and the immune system of the patient can also contribute to bone loss. A conventional approach to treatment is insufficient to address the systemic impairment in bone microstructure, making it crucial to develop a new strategy for understanding osteoporosis [12]. Analysing proteomes can provide insight into patients’ pathophysiological status [13], which is particularly relevant given the observed link between pro-inflammatory states and fractures that are associated with an accelerated decrease in bone mineral density BMD [14, 15].

Chaput et al. [16] found three significant differences between osteoporosis and osteoarthritis (OA) in middle-aged women. In The Osteoporotic Fractures in Men Study, Nielson CM et al. [15] found an association between five proteins and incident hip fracture. When performing proteomic analyses on the osteoporotic population, the comparison population is usually patients with OA [17] due to the ease of obtaining bone tissue. Additionally, there are similarities and even overlaps between risk factors [18, 19] and an inverse relationship between hip fractures and hip OA [20]. In this overlap context, immunology biomarkers that enable differentiation between inflammation in bone (OP) and joint (OA) represent an encouraging possibility for the diagnosis and prognosis of osteoarticular diseases [21]. Even more, the role of immune system in the pathophysiology of osteoporosis [22] suggest that immune dysregulation can trigger inflammatory conditions that negatively affect bone integrity [23]. Even in the acute phase, both hip fracture and hip replacement show a similar elevation of acute phase factors [24, 25]. Therefore, proteomic analyses can aid in understanding the pathophysiology of osteoporosis, the different with other chronic autoimmune rheumatic diseases and lead to the development of more effective treatment strategies.

Insufficient understanding of the pathophysiological and molecular mechanisms of OP and other chronic bone conditions has led to the lack of mechanism-based diagnoses [13]. However, proteomic approaches that examine changes in biomarkers show promise in developing minimally invasive diagnostic biomarkers for OP. Unfortunately, data from older adults are scarce, emphasizing the need to identify valid biomarkers for both diagnosing and evaluating treatments and interventions.

More studies are required to address the knowledge gap concerning the activated molecular mechanisms in OP and to identify potential biomarkers, including aspects of the clinical presentation. In this cross-sectional study, we used a targeted proteomic approach to examine the relationship between immunology biomarker profiles, fracture status, and fracture risk. Our primary aim was to compare immunology biomarker profiles between two patient groups: those with hip OA who were candidates for hip arthroplasty and those with hip fracture who were also candidates for hip arthroplasty. Subsequently, we investigated the association between these profiles and fracture risk, as determined using the FRAX reference tool (as the most extensively risk assessment tool).

## Materials and methods

### Patients and study design

This observational, cross-sectional study scrutinized patients who were referred to the Orthopedic Clinics and Traumatology Services at the University Hospital of Navarre (Pamplona, Spain) between March and October 2021. The criteria for participant inclusion were age ≥ 70 years, a diagnosis of osteoarthritis of the hip being a candidate for hip arthroplasty, a diagnosis of subcapital hip fracture being a candidate for hip arthroplasty, and spinal anaesthesia as the elective technique. The diagnosis of hip OA was based on the criteria of the American College of Rheumatology [26]. Exclusion criteria were diseases that cause secondary OP (e.g., glucocorticoid-induced osteoporosis, rheumatoid arthritis, and autoimmune diseases), terminal illness (advance stages pathologies and cancer) or refusal to participle in the study. We screened 256 older adults, with 83 meeting the inclusion criteria. In our selection process, 112 individuals were excluded due to secondary osteoporosis, 48 due to terminal illnesses, and 13 owing to their refusal to provide informed consent. Consequently, a final cohort of 40 participants was selected for the study, while an additional 43 were excluded. The main reason for exclusion at this point was the change of the day of surgery, which did not allow for the collection and processing of samples. The study flowchart is shown in Appendix A.3. The participants were classified into two groups: hip OA candidates for hip arthroplasty (n = 20) and hip fracture candidates for hip arthroplasty (n = 20). The study received approval from the Institutional Review Board of the University Hospital of Navarre (Pamplona, Spain), under the approval reference PI_2020/125. Every participant involved in the study furnished written informed consent prior to their inclusion in the research.

### Clinical and functional parameters

A comprehensive medical assessment was performed including comorbidities (Cumulative Illness Rating Scale for Geriatrics, CIRS-G) [27], osteoporotic treatments and polypharmacy (defined as regular use of at least five medications). Functional status was assessed by the Barthel index [28], pre-intervention mobility by the FAC (Functional Ambulation Classification) [29] scale, and frailty status by the FRAIL scale [30]. We used pre-fracture values as baseline points. Handgrip strength was measured as part of the Groningen Fitness Test for the Elderly [31] using a Jamar Hydraulic Hand Dynamometer on the day of the surgery. The best of three attempts (with 30 s rest between each attempt) was recorded [32]. Nutritional assessment was performed by body mass index (BMI) calculation (weight/height^2^), and by completing the Mini-nutritional Assessment (MNA) tool [33]. Cognitive status was assessed by Pfeiffer’s Short Portable Mental State Questionnaire (SPMSQ) [34] and depression symptoms were assessed using the Geriatric Depression Scale (GDS-15) [35].

FRAX was determined by factors such as age, BMI, and a set of binary risk elements. These elements included prior fragility fracture, whether a parent has had a hip fracture, current smoking habits, long-term oral glucocorticoid usage, presence of rheumatoid arthritis, other underlying conditions leading to osteoporosis, and alcohol intake. Femoral neck BMD was inputted when it was possible [4].

### Bone mineral density and body composition by dual-energy X-ray absorptiometry (DXA)

BMD and body composition were assessed using dual X-ray absorptiometry (Lunar iDXA, GE Healthcare) one month after surgery. BMD was measured in the total hip, femur neck, posterior-anterior spine, and forearm [36]. Lean mass was measured as Appendicular Skeletal Muscle Mass (ASM) adjusted for height squared (Appendicular Skeletal Muscle Mass Index or ASMI), or body mass index (ASM/BMI) [37].

### Blood extraction and analysis

On the morning of the intervention, fasting peripheral venous blood (PVB) samples were procured from the antecubital vein of the participants. Blood was inverted five times and allowed to sit for 30 min for clotting. Samples were then centrifuged at 2,000 × g for 10 min at 4 °C to obtain plasma and acellular supernatant. Serum aliquots were stored at − 80 °C until use. In order to investigate the viability of utilizing this technology for biomarker analysis, we conducted an assessment of the technical performance of Olink Proteomics’ high-throughput, multiplex proximity extension assays (PEA), specifically the Target 48 Cytokine Panel, for protein screening purposes [38]. The panels had a positive correlation with other established technologies [39]. This emerging technology, developed by Olink Proteomics (Uppsala, Sweden), integrates quantitative real-time Polymerase Chain Reaction (qPCR) with multiplex immunoassays. Essentially, PEA is predicated on dual recognition of a targeted biomarker via a pair of antibodies, each labelled with unique DNA oligonucleotides. These biomarker-specific DNA ‘barcodes’ are quantified using microfluidic qPCR, which allows for high-throughput relative quantification of as many as 1161 human plasma proteins with a minimal volume of biofluids (1 µL suffices for the quantification of 92 biomarkers). The requirement for highly specific antibodies and the employment of target-designed primers augment the specificity and sensitivity of the assays in biological samples. These characteristics, coupled with the utilization of multiple internal controls that monitor each step of the reactions, help to avert unspecific events and minimize background noise [38]. Comprehensive details about PEA technology, its performance, and validation data can be obtained from the manufacturer’s website (www.olink.com) and the biomarkers are listed in Appendices A.1 and B.

The collected data were presented in standard units (pg/mL). For quality, a four-parameter logistic (4PL) curve was generated for the standard curve during product development. Within the limits of quantification (LOQ), the 4PL fitting described the standard curve well with high precision and accuracy, and the concentration could be correctly estimated. Beyond LOQ, the precision and accuracy of the 4PL fitting exhibited a decrease. Cytokine values that fell within the lower and upper limits of quantification (LLOQ and ULOQ, respectively) for each assay – parameters defined during the panel’s development – were not incorporated into the analysis. In total, seven cytokines for which more than 35% of the values were below the limits of detection (LOD) were excluded from all analyses (grey-shaded biomarkers in Appendix A.1).

### Statistical analysis

Background data were tested for normality using the Shapiro-Wilk method. Consequently, the non-parametric (Mann–Whitney U) or parametric (independent t-test) test was used to compare between groups (hip fracture cases *versus* controls) regarding the baseline characteristics in continuous variables. For dichotomous or nominal variables, *Fisher’s* exact or *Pearson X*^2^ were used. Data are presented as mean and standard deviation (SD) if not stated otherwise. The statistical package used to calculate group differences was SPSS version 26 (International Business Machines Corporation [IBM], Armonk, New York, USA). A two-tailed P-value of < 0.05 was considered significant.

**Fig. 1:**
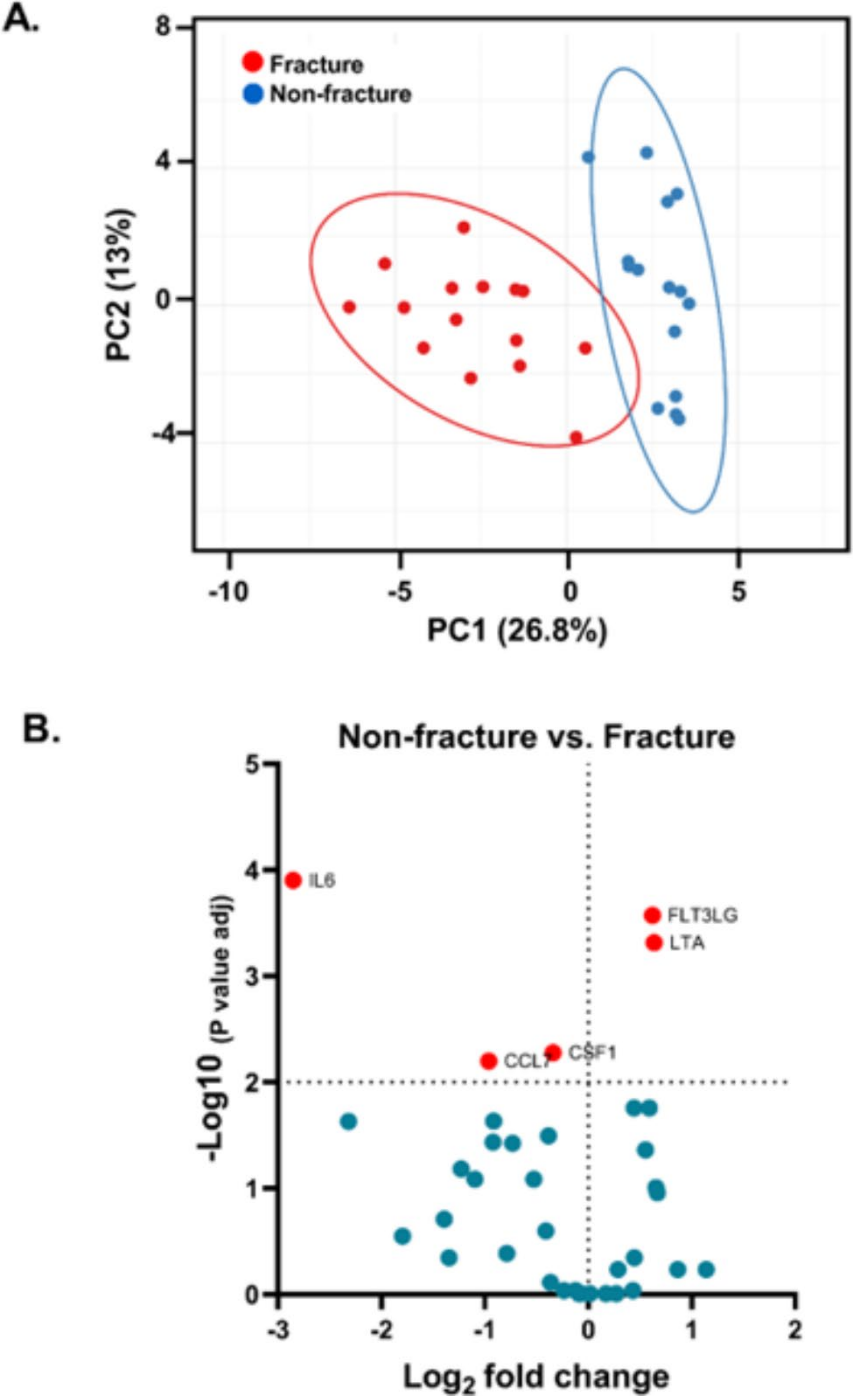
Principal component (PCA) and volcano plot analysis. Panel **A**, Principal component analysis (PCA) between the study groups. The ellipses show a probability of 95% that a new data point from the same group is located inside the ellipse. The red points correspond to fracture subjects, and the blue points correspond to non-fracture subjects. Panel **B**, Volcano plot of the paired t-test between non-fracture vs. fracture. Statistically significant differences in protein expression levels were found after correction with Benjamini–Hochberg, which is represented by all the proteins being presented as red dots, that is, the corrected p‐values did reach < 0.05. The dotted line represents the corrected significance threshold of 0.05. On the y‐axis are log_10_ of p‐values and on the x‐axis is the log_2_ fold change between the two groups where a positive fold change indicates a lower protein level in the non-fracture than in the fracture

We used *Tukey’s* fences method to detect observations out of the normal range by using interquartile ranges [40], which are often used for detecting outliers in various fields [41]. 55 outliers were excluded from the analysis out of the 1800 values analyzed using the Olink platform. Before performing Tukey’s fences, the normality of the data was checked before fitting the curve. Features with > 70% missing values in the real samples or > 10% outlier values in the serum samples were deleted first, and 36 biomarkers passed quality control (Appendix B). Serum biomarkers in pg/mL values were analyzed using two unpaired t-tests, *Benjamini–Hochberg* method for *p-*value correction with a 5% false discovery rate, and a distribution boxplot. *P* values < 0.05 were considered statistically significant after correction with the *Benjamini–Hochberg* method. Principal component analysis and Volcano plot (Fig. 1) assessed the distribution groups, using singular value decomposition with imputation (pre-normalized data, no transformation), and visualized using ClustVis [42]. R-squared and goodness-of-fit measure for linear regression models was calculated including the clinical variables and significant biomarkers related to fracture risk (FRAX hip and major fracture). After these analyses, a one-way analysis of covariance (ANCOVA) was performed adjusted for age, sex, body mass index, and FRAX (hip and major) score with effect size of fracture vs. non-fracture. These analyses were performed using GraphPad Prism 9 program for Windows. Protein–protein association network analysis was created using the online database tool STRING version 11 [43]. Protein accession numbers (UniProt) from significant proteins were entered in the search engine (multiple proteins) with the following parameters: Organism *Homo sapiens*, the maximum number of interactions was query proteins only, interaction score was set to medium confidence (0.400), and an FDR of ≤ 0.01 was used when classifying the Biological Process (GO) of each protein.

## Results

### Baseline characteristics

We provided an overview of the demographic, clinical, and functional features of the patients included in the analysis (Table 1). The study included 40 older adults (72.5% female) with a mean age (SD) of 81.23 (8.23) years. As clinically expected, the scores for BMI, functional status, FRAX scores, bone mineral density and body composition parameters were all significantly lower in the fracture group than in the non-fracture group (p < 0.05).

**Table 1:** Demographic, clinical, and functional characteristics of the patients included for analysis (values expressed as mean and standard deviation unless otherwise specified)

	Full sample (n = 40)	Fracture group (n = 20)	Non-fracture group (n = 20)	P value^*^
*Demographic*				
Age, years	81.23 (8.23)	87.25 (6.73)	75.20 (4.15)	**0.026**
Sex (men/female), n (%)	11 (27.5)/29 (72.5)	4 (20)/16 (80)	7 (35)/13 (65)	0.480
BMI (kg/m^2^)^a^	27.39 (4.72)	24.91 (2.74)	29.87 (5.02)	**0.003**
***Clinical status***				
CIRS-G score	11.45 (4.21)	12.7 (4.81)	10.2 (3.17)	0.060
Polypharmacy score	6.28 (3.16)	7.25 (3.09)	5.3 (3)	0.534
Osteoporosis (n, %)	10 (25%)	4 (20%)	6 (30%)	0.716
***Functional status***				
Barthel Index (ADL), score^c^	81.63 (26.13)	67.5 (30.41)	95.75 (7.48)	**< 0.001**
Functional Ambulation Category (n, %)				
FAC 0 to 1	3 (7.5%)	3 (15%)	0 (0)	**0.032**
FAC 4 to 5	36 (92.5%)	17 (85%)	20 (100%)	
Frailty score^d^	2.18 (1.69)	3.05 (1.47)	1.3 (1.42)	**< 0.001**
Hand grip strength (Kg)	17.63 (9.8)	11.3 (6.24)	23.95 (8.6)	**< 0.001**
MNA score^e^	23.43 (6.51)	18.83 (6.08)	28.03 (2.33)	**< 0.001**
Pfeiffer’s SPMSQ^f^	2.55 (3.80)	5.05 (4.05)	0.5 (0.224)	**< 0.001**
Depression score (n, %)^g^	8 (20%)	6 (42.9%)	2 (10%)	**0.026**
FRAX mayor score^h^	9.76 (7,15)	13.4 (6.99)	6.12 (5.29)	**< 0.001**
FRAX hip score^i^	4.43 (3.85)	6.29 (3.79)	2.58 (2.94)	**< 0.001**
***Bone mineral density and body composition***				
BMD^j^ - total hip	0.873 (0.186)	0.735 (0.079)	0.976 (0.177)	**0.001**
BMD – femoral neck	0.869 (0.211)	0.739 (0.119)	0.966 (0.217)	**0.011**
BMD – lumbar spine	1.153 (0.256)	0.981 (0.18)	1.239 (0.247)	**0.007**
BMD – foreman	0.768 (0.314)	0.679 (0.127)	0.812 (0.37)	0.281
ASMI^k^	6.24 (1.63)	5.06 (1.27)	7.43 (0.95)	**< 0.001**
ASM/BMI^l^	0.607 (0.188)	0.526 (0.155)	0.687 (0.187)	**0.005**

^a^BMI (body mass index)

^b^The Cumulative Illness Rating Scale for Geriatrics (CIRS-G) scale evaluates individual body systems, ranging from 0 (best) to 56 (worst)

^c^The Barthel Index ranges from 0 (severe functional dependence) to 100 (functional independence)

^d^Frail Scale ranges from 0 to 5 and indicates frailty with ≥ 3

^e^Mini-Nutritional Assessment (MNA).

^f^Pfeiffer’s Short Portable Mental State Questionnaire (SPMSQ) ranges errors from 0 (best) to 10 (worst)

^g^The Geriatric Depression Scale (GDS-15) ranges from 0 to 15 and indicates symptomatic depression with ≥ 5

^h^FRAX 10-year fracture probability of mayor osteoporotic fracture (%). Mean and SD

^i^FRAX 10-year fracture probability of hip fracture (%)

^j^BMD (bone mineral density, g/cm^2^)

^k^ASMI (Appendicular Skeletal Muscle Index, kg)

^l^ASM/BMI (Appendicular lean mass adjusted for BMI).

* p-value for different groups in percentage (Pearson X^2^, expect no normal distribution; Fisher’s exact test) or means (t-student, expect no normal distribution; U de Mann-Whitney). The bold values are statistically significant

### Principal component analysis, Volcano plot and protein association network analysis

A score plot was generated to show the separation between the fracture and non-fracture groups. The principal component analysis did not reveal any abnormal deviations between the two groups (Fig. 1A) with a very similar pattern within the same group and differences between them. The outcome obtained using this selection criterion is presented in the volcano plot displayed in Fig. 1B. It was possible to isolate five biomarkers that showed high differentiation between the study groups.

**Fig. 2:**
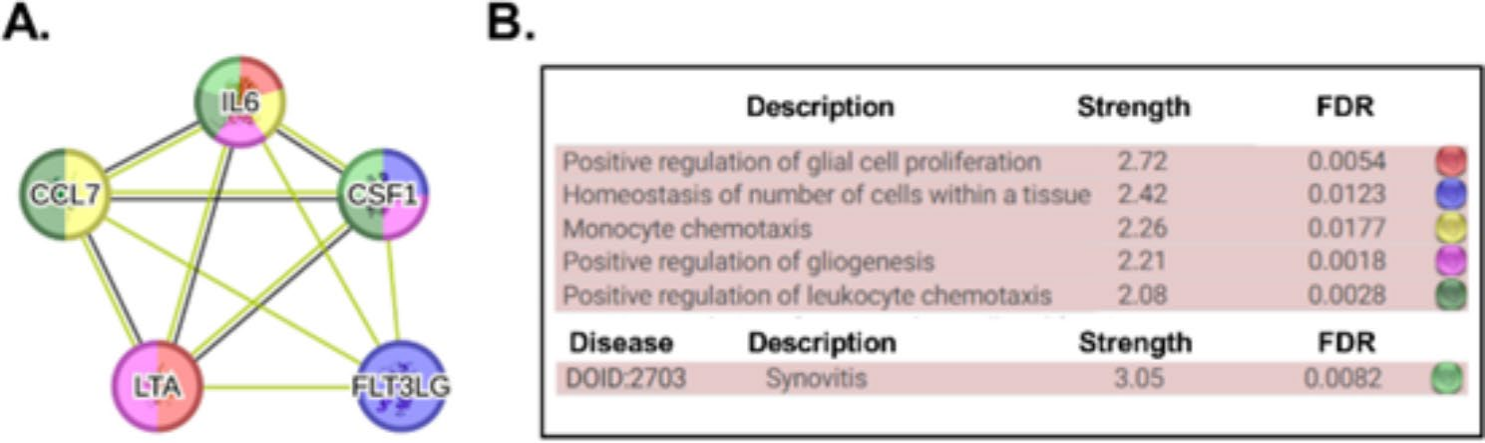
Pathway analysis of immunology proteins associated with the metabolic process in bone. Functional protein network analysis of significant proteins associated with metabolic process. The STRING version 11 was used to create the network analysis (https://string-db.org/). In the network, each protein is represented by a coloured node, and protein–protein interaction and association are represented by an edge visualized as a coloured lined (type of interaction). Known interactions used were from curated databases (turquoise) and experimentally determined (pink). Predicted interactions were gene neighbourhood (green), gene fusion (red) and gene-co-occurrence (dark blue), and other interactions were text mining (yellow), coexpression (black), and protein homology (purple). Interleukin 6 (IL-6), Lymphotoxin-alpha (LT-α) or tumor necrosis factor-beta (TNF-β), Fms-related tyrosine kinase 3 ligand (FLT3LG), Colony stimulating factor 1 (CSF1), also known as macrophage colony-stimulating factor (M-CSF), and Chemokine (C-C motif) ligand 7 (CCL7)

Changes were observed in the five proteins included: Interleukin 6 (IL-6), Lymphotoxin-alpha (LT-α) or tumor necrosis factor-beta (TNF-β), Fms-related tyrosine kinase 3 ligand (FLT3LG), Colony stimulating factor 1 (CSF1), also known as macrophage colony-stimulating factor (M-CSF), and Chemokine (C-C motif) ligand 7 (CCL7). Enrichment analysis with multiple testing corrections was used to assign related gene categories to their associated pathways using gene ontology (summarized in Fig. 2).

### Biomarkers difference and correlation with fracture risk

**Fig. 3:**
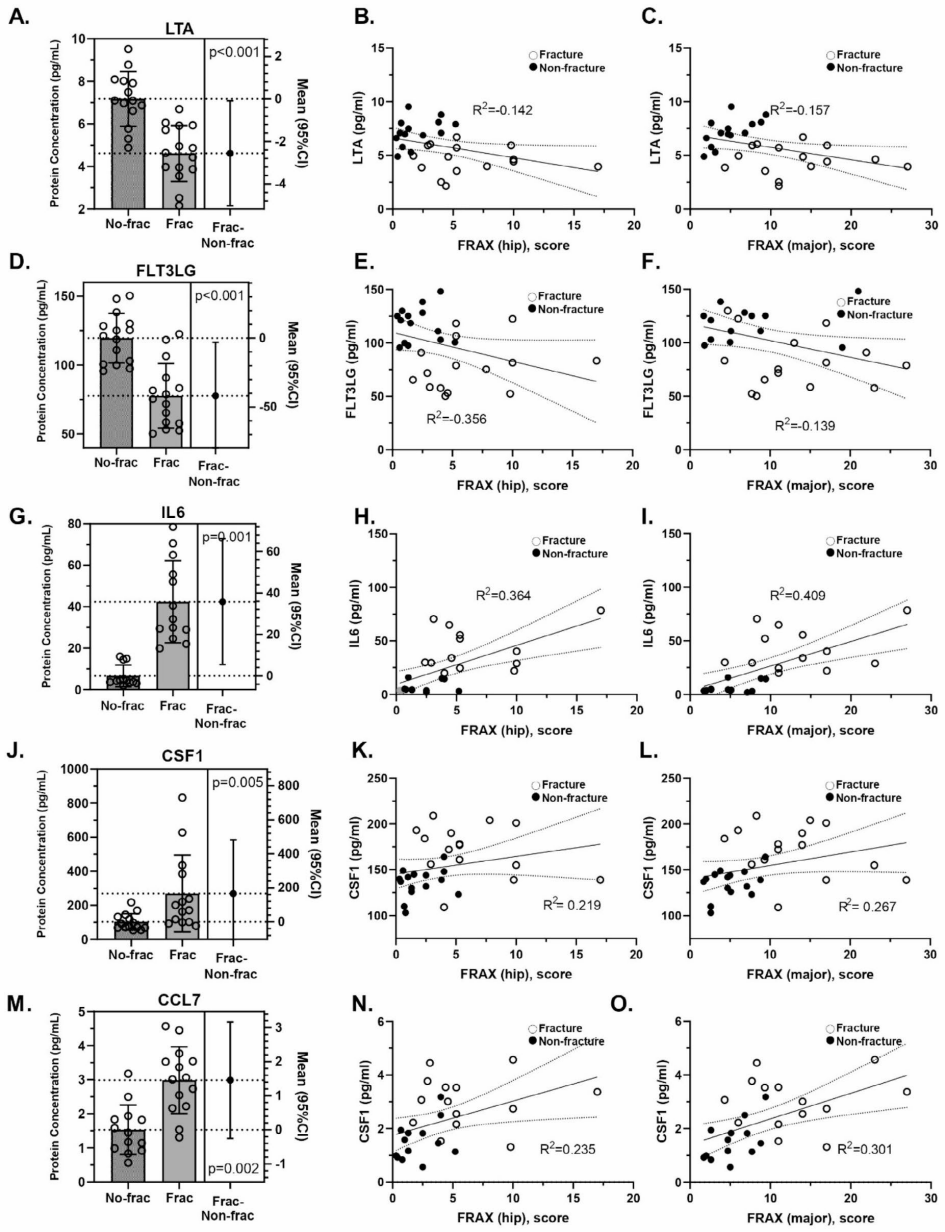
Group difference (fracture vs. non-fracture) and their association with FRAX (hip and major) score with significant plasma biomarkers. Panel **A**, **D**, **G**, **J** and **M** show mean plots of the five proteins with the most significant changes in protein expression levels following t-tests between fracture vs. non-fracture groups. Panel **B**, **C**, **E**, **F**, **H**, **I**, **K**, **L**, **N**, and **O** figures, show the lineal regression between fracture vs. non-fracture groups with FRAX (hip and major) scores with significant plasma biomarkers. Solid lines: estimation; dashed curved lines: 95% confidence interval limits. Lymphotoxin-alpha (LT-α) or tumor necrosis factor-beta (TNF-β), Fms-related tyrosine kinase 3 ligand (FLT3LG), Interleukin 6 (IL-6), Colony stimulating factor 1 (CSF1), also known as macrophage colony-stimulating factor (M-CSF), and Chemokine (C-C motif) ligand 7 (CCL7)

After conducting two unpaired t-tests with the *Benjamini-Hochberg* method for p-value correction, it was found that these five cytokines were significantly different between fracture and non-fracture patients (p < 0.05). The mean plots in Fig. 3A, D, G, J, and M display the levels of these five proteins. LT-α and FLT3LG were found to be higher in non-fracture patients, whereas IL-6, CSF1, and CCL7 were found to be higher in fracture patients. (Appendix A.2) shows the immunology biomarkers that were not found to be significantly associated with fracture status.

Furthermore, linear regression models showed moderate (*R*^2^ = 0.409) but significant (p = 0.001) positive correlations between IL-6 levels and the risk of major fracture, as shown in Fig. 3I. The levels of CSF1 (*R*^2^ = 0.267; p = 0.005) and CCL7 (*R*^2^ = 0.301; p = 0.002) had a weak correlation with the risk of fracture. On the other hand, LTA (*R*^2^=-0.157; p < 0.001) and FLT3LG (*R*^2^=-0.139; p < 0.001) exhibited a negative relation with the risk of fracture.

After the ANCOVA was performed adjusted for age, sex, body mass index, and FRAX (hip and major) score and with effect size of fracture vs. non-fracture, all immunology biomarkers maintained significant (p < 0.05) expect for CSF1 (Appendix A.4).

## Discussion

This cross-sectional study utilized a targeted proteomic approach to identify potential biomarkers of hip fracture in older adults. The study identified five potential biomarkers, namely serum IL-6, CSF1, LT-α, FLT3LG, and CCL7, which may have significant implications for fracture risk. Out of these biomarkers, three (IL-6, CSF1, and CCL7) exhibited a positive relationship with fracture risk based on the FRAX reference tool, while two (LT-α and FLT3LG) had a negative relationship with fracture risk. While previous evidence has suggested an association between biomarkers and osteoporosis [23, 44], this study is the first to examine the relationship between FRAX and serum cytokines. These findings have the potential to pave the way for developing effective biomarker-based diagnostic tools and interventions for osteoporosis, which could significantly improve clinical outcomes for older adults at risk of hip fracture.

In this study, we utilized PEA to characterize serum cytokines related to signaling and inflammatory processes in older adults with hip fractures compared to other adults undergoing elective orthopedic surgery. Given the multitude of immunology biomarkers that are altered in rheumatic diseases [45], the choice of OA as the control group in this study allows us to confirm the association of these five biomarkers with OP [21], ruling out their association with OA as other most prevalent rheumatic disease in the older population. There are some similarities between osteoporosis (OP) and osteoarthritis (OA) [18–21], the characteristics of these groups are quite different due to factors such as age [46] and the presence of risk factors. As observed in our study and supported by existing literature, patients with OP and hip fractures are notably older [25, 46, 47] and often in a poorer nutritional state [48]. This age and nutritional disparity can inherently influence the outcomes of studies involving these populations. For instance, underweight is a risk factor for OP [49, 50] and while obesity stimulates the development of OA [19, 50] and maybe acts as OP protector factor [51]. Additionally, functional capacity is an independent factor for hip fracture [52], whereas hip arthroplasty is a common treatment for OA patients [53].

In this exploratory study, these clinical differences may have contributed to differences in cytokine profiles, which highlights the need for closer case-control clinical matching in further studies. Our interpretation of the functional mechanisms of the five identified proteins is that they are involved in immune and inflammatory processes. While these proteins have traditionally been associated with synovial membrane inflammation (synovitis), recent findings in osteoimmunology suggest that immune dysregulation can trigger inflammatory conditions that negatively affect bone integrity [23]. These findings may have important implications for understanding the complex interplay between inflammation and bone health in older adults.

Studying the molecules reported in this study is important because low-grade inflammation is a key factor in the pathogenesis of various widespread diseases, particularly osteoporosis [54]. Although it is not yet understood how circulating peptides reflect activity in musculoskeletal tissues, inflammatory mediators such as reactive oxygen species (ROS), pro-inflammatory cytokines, and chemokines directly or indirectly affect bone cells and contribute to the development of osteoporosis [15, 44]. Prior endeavors have concentrated on the identification of prospective biomarkers capable of prognosticating the likelihood of osteoporosis, either as standalone predictors or in conjunction with clinical risk factors and BMD.

The biomarkers identified in this study have been previously investigated concerning osteoporosis. For example, increased levels of IL-6 induce osteoclastogenesis, the accumulation of T-cells (Th17), and the production of RANKL, which promotes bone resorption [23]. IL-6 also upregulates bone destruction by releasing protease enzymes from inflammatory cells [44]. Even though the expression of RANKL in an array of cell types, including osteoblasts, research suggests that osteocytes predominantly contribute to the pool of RANKL essential for osteoclast genesis [55].

Despite the positive associations found between IL-6 and fracture risk (*R*^2^ = 0.409 for major fracture risk, and R^2^ = 0.364 for hip fracture risk), it is currently unclear whether blood IL-6 concentration can accurately predict fracture risk.

LT-α, also known as tumor necrosis factor-beta (TNF-β), is a cytokine belonging to the tumor necrosis factor superfamily that mediates a range of inflammatory, immunostimulatory, and antiviral responses [56]. Although involved in the genesis and treatment of osteoarthritis [57], it induces osteoclastogenesis alongside RANKL [58]. However, when TNF- α is present in abundance, studies suggest that its role is secondary to that of TNF- α [59]. The significant but weak (*R*^2^ = − 0.157 in the best case) correlation with the control group may be due to its relationship with both processes and its secondary role.

FLT3LG is a hematopoietic cytokine related to growth factors that increase the number of immune cells by activating hematopoietic progenitors. FLT3LG studies in the biomedical literature are more related to leukaemia than musculoskeletal diseases [60]. The role of this cytokine in bone joints is debated and has mainly been described in rheumatoid arthritis, where it is considered to be a negative regulator of osteoclastogenesis and a bone-protective factor [61]. This may explain the weak association with fracture risk seen in our study (*R*^2^ = − 0.356).

CSF1, also known as macrophage colony-stimulating factor (M-CSF), is a secreted cytokine that causes hematopoietic stem cells to differentiate into macrophages or other related cell types. CSF1 is involved in multiple functions throughout the body, including bone health. In bone, stromal cells secrete CSF1, which affects T-cell differentiation in osteoclastogenesis [23]. CSF1 is crucial for the proliferation, differentiation, and motility of osteoclasts [62], making it a key therapeutic target for osteoporosis [63]. In our study, we found that CSF1 levels were different between the fracture and control groups (p = 0.005), but with a weak correlation to fracture risk. Despite its biological plausibility, CSF1 did not retain its significance after adjusting for multiple confounders, likely due to the sample size. While it was adequate for initial observations, it might not have been sufficiently large to detect subtle effects of CSF1 once other variables were taken into account.

CCL7 belongs to the CC chemokine family and its role in osteoporosis is currently under study [64]. RANKL induces the expression of many chemokines including CCL7, to enhance osteoclast formation. Currently, CCL7 is being studied as a potential target for postmenopausal osteoporosis [65]. Our findings support the relationship with OP (p = 0.002), with a weak correlation with fracture risk.

Despite the importance of cytokines in bone regulation, other cytokines related to bone loss, such as IL-1B, IFNG, and TNF, did not show significance in our study [23, 44]. Considering the widely acknowledged limitations of utilizing BM) in the evaluation of fracture risk within the bone health research community, there is an ongoing pursuit to discover and validate novel biomarkers for clinical application. This endeavor stems from the growing understanding of bone regulation, which contributes to an expanding pool of knowledge in the field. Our findings suggest that the weak association of IL-6, CSF1, and CCL7 with fracture risk may be related to the implications of these cytokines in inflammaging and other age-related diseases [66] in older adults with high comorbidity burden (especially OA [67]) and polypharmacy [68, 69]. The lack of differences in these cytokines may be due to similar inflammaging-related characteristics between the study groups. Hence, based on the current body of evidence, the utilization of these three prospective biomarkers as predictors of treatment responses to novel anti-osteoporotic medications is not supported [70].

The main strength of this exploratory analysis is its potential to provide a new tool for estimating an individual’s risk of experiencing a hip fracture or a major osteoporotic fracture based on serum analysis, which could guide clinical decision-making and assist healthcare professionals in identifying individuals who may benefit from interventions to reduce their risk of fractures. The development of serum biomarkers for fracture risk in older adults is of interest in clinical practice due to the association of fractures with disability, premature mortality, and increased utilization of medical resources [3]. Moreover, Olink Proteomics’ high-throughput allows for reliable analysis of these very low values of immunology biomarkers, such LTA and CCL7 (with levels < 10pg/ml) but these results should be taken with caution.

However, it is essential to recognize and consider the limitations of our study. First, the analysis was cross-sectional, meaning causative relationships cannot be considered. Longitudinal studies will be necessary to determine the temporal relationship between changes in cytokine profiles and the development of a hip fracture. Second, the small study population comprised only Caucasians, so our findings cannot be generalized to other ethnic groups and limited the statistical strength (specially for CSF1). Additionally, although the cohort was extensively characterized, it was relatively small, and analyses involved a large set of variables. The two comparison groups were not closely matched in terms of demographic or clinical characteristics, which may have confounded our results, but after adjusted for age, sex, body mass index, and FRAX score; most of them were still significant different.

## Conclusion

To summarize, our cross-sectional study identified five immunology biomarkers (IL-6, CSF1, LT-α, FLT3LG and CCL7) that were associated with hip fracture and have potential correlation with fracture risk. This study provides a potential contribution by highlighting immunology biomarkers that could be further studied to estimate fracture risk and potentially delay the onset of osteoporosis and fragility fractures in older adults. However, to increase the clinical relevance of these biomarkers and small sample, validation and replication in longitudinal cohorts with diverse populations are needed.

### Electronic supplementary material

Below is the link to the electronic supplementary material.

Supplementary Material 1

Supplementary Material 2

Supplementary Material 3

## Data Availability

All data relevant to the study are included in the article or uploaded as supplementary information.

## Abbreviations

ASM: Appendicular Skeletal Muscle Mass
ASMI: Appendicular Skeletal Muscle Mass Index
BMD: Bone mineral density
BMI: Body mass index
BMT: Bone turnover markers
CCL7: Chemokine (C-C motif) ligand 7
CIRS-G: Cumulative Illness Rating Scale for Geriatrics
CSF1: Colony stimulating factor 1
DXA: Dual-Energy X-ray Absorptiometry
FLT3LG: Fms-related tyrosine kinase 3 ligand
IL-6: Interleukin 6
LT-α: Lymphotoxin-alpha
LOQ: Limits of Quantification
MNA: Mini-nutritional Assessment
M-CSF: Macrophage colony-stimulating factor
OA: Osteoarthritis
OP: Osteoporosis
PEA: Proximity extension assay
PVB: Fasting peripheral venous blood
ROS: Reactive oxygen species
SPMSQ: Pfeiffer’s Short Portable Mental State Questionnaire
TNF-β: Tumor necrosis factor-beta

## References

[1] Kanis JA, Cooper C, Rizzoli R, Reginster JY (2019). European guidance for the diagnosis and management of osteoporosis in postmenopausal women. Osteoporos Int.

[2] Borgström F, Karlsson L, Ortsäter G, Norton N, Halbout P, Cooper C et al. Fragility fractures in Europe: burden, management and opportunities. Arch Osteoporos. 2020;15. 10.1007/s11657-020-0706-y PMC7166207 32306163

[3] Al Saedi A, Feehan J, Phu S, Duque G (2019). Current and emerging biomarkers of frailty in the elderly. Clin Interv Aging.

[4] Kanis JA, Johansson H, Harvey NC. McCloskey E V. A brief history of FRAX. Archives of Osteoporosis. 2018;13. 10.1007/s11657-018-0510-0 PMC6290984 30382424

[5] Leslie WD, Majumdar SR, Morin SN, Lix LM (2015). Why does rate of bone density loss not predict fracture risk?. J Clin Endocrinol Metab.

[6] Lorentzon M, Branco J, Brandi ML, Bruyère O, Chapurlat R, Cooper C (2019). Algorithm for the use of biochemical markers of bone turnover in the diagnosis, Assessment and Follow-Up of treatment for osteoporosis. Adv Ther.

[7] El Miedany Y (2020). FRAX: re-adjust or re-think. Archives of Osteoporosis.

[8] Sheehan KJ, Williamson L, Alexander J, Filliter C, Sobolev B, Guy P (2018). Prognostic factors of functional outcome after hip fracture surgery: a systematic review. Age Ageing.

[9] Sun X, Chen Y, Gao Y, Zhang Z, Qin L, Song J (2022). Prediction models for osteoporotic fractures risk: a systematic review and critical Appraisal. Aging and Disease.

[10] Wu Q, Xiao X, Xu Y. Performance of FRAX in predicting fractures in US postmenopausal women with varied race and genetic profiles. J Clin Med. 2020;9. 10.3390/jcm9010285 PMC7019759 31968614

[11] Vandenput L, Johansson H, McCloskey EV, Liu E, Åkesson KE, Anderson FA (2022). Update of the fracture risk prediction tool FRAX: a systematic review of potential cohorts and analysis plan. Osteoporos Int.

[12] Rachner TD, Khosla S, Hofbauer LC (2011). Osteoporosis: now and the future. Lancet (London England).

[13] Zhang H, Recker R, Lee WNP, Xiao GG (2010). Proteomics in bone research. Expert Rev Proteomics.

[14] Sponholtz TR, Zhang X, Fontes JDT, Meigs JB, Cupples LA, Kiel DP (2014). Association between inflammatory biomarkers and bone mineral density in a community-based cohort of men and women. Arthritis Care Res.

[15] Nielson CM, Wiedrick J, Shen J, Jacobs J, Baker ES, Baraff A (2017). Identification of hip BMD loss and fracture risk markers through Population-Based serum proteomics. J Bone Miner Res.

[16] Chaput CD, Dangott LJ, Rahm MD, Hitt KD, Stewart DS, Sampson HW (2012). A proteomic study of protein variation between osteopenic and age-matched control bone tissue. Exp Biol Med.

[17] Rollín R, Marco F, Camafeita E, Calvo E, López-Durán L, Jover JÁ (2008). Differential proteome of bone marrow mesenchymal stem cells from osteoarthritis patients. Osteoarthr Cartil.

[18] Im G, Il, Kim MK (2014). The relationship between osteoarthritis and osteoporosis. J Bone Miner Metab.

[19] Bultink IEM, Lems WF. Osteoarthritis and osteoporosis: what is the overlap? Curr Rheumatol Rep. 2013;15. 10.1007/s11926-013-0328-0 23508809

[20] Franklin J, Englund M, Ingvarsson T, Lohmander S (2010). The association between hip fracture and hip osteoarthritis: a case-control study. BMC Musculoskelet Disord.

[21] Nagy E, Nagy-Finna C, Popoviciu H-V, Kovács B (2020). Soluble biomarkers of osteoporosis and osteoarthritis, from pathway mapping to clinical trials: an update. Clin Interv Aging.

[22] Zhang W, Gao R, Rong X, Zhu S, Cui Y, Liu H et al. Immunoporosis: role of immune system in the pathophysiology of different types of osteoporosis. Front Endocrinol (Lausanne). 2022;13. 10.3389/fendo.2022.965258 PMC9487180 36147571

[23] Ahmad SS, Ahmed F, Ali R, Ghoneim MM, Alshehri S, Najmi AK (2022). Immunology of osteoporosis: relevance of inflammatory targets for the development of novel interventions. Immunotherapy.

[24] Pertusa C, Tarín JJ, Cano A, García-Pérez MÁ, Mifsut D (2021). Serum microRNAs in osteoporotic fracture and osteoarthritis: a genetic and functional study. Sci Rep.

[25] Sedlář M, Kudrnová Z, Trča S, Mazoch J, Malíková I, Kvasnička J (2008). Inflammatory response in patients undergoing hip surgery due to osteoarthrosis or different types of hip fractures. Osteoarthr Cartil.

[26] Altman R, Alarcón G, Appelrouth D, Bloch D, Borenstein D, Brandt K (1991). The American College of Rheumatology criteria for the classification and reporting of osteoarthritis of the hip. Arthritis Rheum.

[27] Conwell Y, Forbes NT, Cox C, Caine ED (1993). Validation of a measure of physical illness burden at autopsy: the cumulative illness rating scale. J Am Geriatr Soc.

[28] MAHONEY FI, BARTHEL DW (1965). FUNCTIONAL EVALUATION: THE BARTHEL INDEX. Md State Med J.

[29] MK H, KM G, MR M (1984). L P-B. Clinical gait assessment in the neurologically impaired. Reliability and meaningfulness. Phys Ther.

[30] Morley JE, Malmstrom TK, Miller DK (2012). A simple frailty questionnaire (FRAIL) predicts outcomes in middle aged african americans. J Nutr Heal Aging.

[31] Lemmink KAPM, Han K, De Greef MHG, Rispens P, Stevens M (2001). Reliability of the Groningen Fitness Test for the Elderly. J Aging Phys Act.

[32] Dodds RM, Syddall HE, Cooper R, Benzeval M, Deary IJ, Dennison EM et al. Grip strength across the life course: normative data from twelve british studies. PLoS ONE. 2014;9. 10.1371/journal.pone.0113637 PMC4256164 25474696

[33] Cereda E (2012). Mini nutritional assessment. Curr Opin Clin Nutr Metab Care.

[34] De La Martínez J, Herrero RD, Vilches MCO, Taberné CA, Colomer CA, Luque RL (2001). Cross-cultural adaptation and validation of Pfeiffer’s test (short Portable Mental Status Questionnaire [SPMSQ]) to screen cognitive impairment in general population aged 65 or older. Med Clin (Barc).

[35] de la Martínez J, Onís Vilches MC, Dueñas Herrero R, Albert Colomer C, Aguado Taberné C, Luque Luque R (2002). Versión española del cuestionario de yesavage abreviado (GDS) para el despistaje de depresión en mayores de 65 años: Adaptación y validación. MEDIFAM - Rev Med Fam y Comunitaria.

[36] Lupsa BC, Insogna K (2015). Bone health and osteoporosis. Endocrinol Metab Clin North Am.

[37] Cruz-Jentoft AJ, Bahat G, Bauer J, Boirie Y, Bruyère O, Cederholm T (2019). Sarcopenia: revised european consensus on definition and diagnosis. Age Ageing.

[38] Assarsson E, Lundberg M, Holmquist G, Björkesten J, Thorsen SB, Ekman D et al. Homogenous 96-plex PEA immunoassay exhibiting high sensitivity, specificity, and excellent scalability. PLoS ONE. 2014;9. 10.1371/journal.pone.0095192 PMC3995906 24755770

[39] Petrera A, Von Toerne C, Behler J, Huth C, Thorand B, Hilgendorff A (2021). Multiplatform Approach for plasma proteomics: complementarity of Olink Proximity Extension Assay Technology to Mass Spectrometry-Based protein profiling. J Proteome Res.

[40] Tukey JW (1977). Exploratory data analysis.

[41] Luo J, Frisken S, Machado I, Zhang M, Pieper S, Golland P (2018). Using the variogram for vector outlier screening: application to feature-based image registration. Int J Comput Assist Radiol Surg.

[42] Metsalu T, Vilo J, ClustVis (2015). A web tool for visualizing clustering of multivariate data using principal component analysis and heatmap. Nucleic Acids Res.

[43] Szklarczyk D, Kirsch R, Koutrouli M, Nastou K, Mehryary F, Hachilif R (2023). The STRING database in 2023: protein–protein association networks and functional enrichment analyses for any sequenced genome of interest. Nucleic Acids Res.

[44] Saxena Y, Routh S, Mukhopadhaya A. Immunoporosis: role of Innate Immune cells in osteoporosis. Front Immunol. 2021;12. 10.3389/fimmu.2021.687037 PMC8374941 34421899

[45] Giacomelli R, Afeltra A, Alunno A, Bartoloni-Bocci E, Berardicurti O, Bombardieri M (2019). Guidelines for biomarkers in autoimmune rheumatic diseases - evidence based analysis. Autoimmun Rev.

[46] Kiebzak GM (1991). Age-related bone changes. Exp Gerontol.

[47] Salari N, Darvishi N, Bartina Y, Larti M, Kiaei A, Hemmati M (2021). Global prevalence of osteoporosis among the world older adults: a comprehensive systematic review and meta-analysis. J Orthop Surg Res.

[48] Huang W, Xiao Y, Wang H, Li K. Association of geriatric nutritional risk index with the risk of osteoporosis in the elderly population in the NHANES. Front Endocrinol (Lausanne). 2022;13. 10.3389/fendo.2022.965487 PMC9744963 36523597

[49] Park S-M, Park J, Han S, Jang H-D, Hong J-Y, Han K (2023). Underweight and risk of fractures in adults over 40 years using the nationwide claims database. Sci Rep.

[50] Le Manach Y, Collins G, Bhandari M, Bessissow A, Boddaert J, Khiami F (2015). Outcomes after hip fracture surgery compared with elective total hip replacement. JAMA.

[51] Saarelainen J, Kiviniemi V, Kröger H, Tuppurainen M, Niskanen L, Jurvelin J (2012). Body mass index and bone loss among postmenopausal women: the 10-year follow-up of the OSTPRE cohort. J Bone Miner Metab.

[52] Araiza-Nava B, Méndez-Sánchez L, Clark P, Peralta-Pedrero ML, Javaid MK, Calo M (2022). Short- and long-term prognostic factors associated with functional recovery in elderly patients with hip fracture: a systematic review. Osteoporos Int.

[53] Clausen S, Hartvigsen J, Boyle E, Roos EM, Grønne DT, Ernst MT (2021). Prognostic factors of total hip replacement during a 2-year period in participants enrolled in supervised education and exercise therapy: a prognostic study of 3657 participants with hip osteoarthritis. Arthritis Res Ther.

[54] Portal-Núñez S, de la Fuente M, Díez A, Esbrit P (2016). Oxidative stress as a possible therapeutic target for osteoporosis associated with aging. Rev Osteoporos y Metab Miner.

[55] Nakashima T, Hayashi M, Fukunaga T, Kurata K, Oh-Hora M, Feng JQ (2011). Evidence for osteocyte regulation of bone homeostasis through RANKL expression. Nat Med.

[56] Nedwin GE, Naylor SL, Sakaguchi AY, Smith D, Jarrett-Nedwin J, Pennica D (1985). Human lymphotoxin and tumor necrosis factor genes: structure, homology and chromosomal localization. Nucleic Acids Res.

[57] Blumenfeld I, Livne E (1999). The role of transforming growth factor (TGF)-β, insulin-like growth factor (IGF)-1, and interleukin (IL)-1 in osteoarthritis and aging of joints. Exp Gerontol.

[58] Yao Z, Lei W, Duan R, Li Y, Luo L, Boyce BF (2017). RANKL cytokine enhances TNF-induced osteoclastogenesis independently of TNF receptor associated factor (TRAF) 6 by degrading TRAF3 in osteoclast precursors. J Biol Chem.

[59] Croft M, Siegel RM, Beyond TNF (2017). TNF superfamily cytokines as targets for the treatment of rheumatic diseases. Nat Rev Rheumatol.

[60] Wu M, Li C, Zhu X. FLT3 inhibitors in acute myeloid leukemia. J Hematol Oncol. 2018;11. 10.1186/s13045-018-0675-4 PMC6280371 30514344

[61] Voronov I, Manolson MF, Editorial (2016). Flt3 ligand–friend or foe?. J Leukoc Biol.

[62] Han Y, You X, Xing W, Zhang Z, Zou W. Paracrine and endocrine actions of bone - the functions of secretory proteins from osteoblasts, osteocytes, and osteoclasts. Bone Res. 2018;6. 10.1038/s41413-018-0019-6 PMC5967329 29844945

[63] McDonald MM, Kim AS, Mulholland BS, Rauner M. New Insights into Osteoclast Biology. JBMR Plus. 2021;5. 10.1002/jbm4.10539 PMC8441501 34532619

[64] Hu M, Ding H, Chao R, Cao Z (2023). The hub genes related to osteoporosis were identified by Bioinformatics Analysis. Biomed Res Int.

[65] Yuan S, guo, Hu H ling, Wang Xjia, Yang J, cheng, Zhou R ping, Bai X et al. chun,. Bindarit Reduces Bone Loss in Ovariectomized Mice by Inhibiting CCL2 and CCL7 Expression via the NF-κB Signaling Pathway. Orthop Surg. 2022;14:1203–16. 10.1111/os.13252 PMC9163972 35470579

[66] Chung HY, Cesari M, Anton S, Marzetti E, Giovannini S, Seo AY (2009). Molecular inflammation: underpinnings of aging and age-related diseases. Ageing Res Rev.

[67] Yang L, Chen Z, Guo H, Wang Z, Sun K, Yang X (2021). Extensive cytokine analysis in synovial fluid of osteoarthritis patients. Cytokine.

[68] Franceschi C, Campisi J (2014). Chronic inflammation (inflammaging) and its potential contribution to Age-Associated Diseases. Journals Gerontol Ser A.

[69] McConnell M, Shieh A. Polypharmacy in osteoporosis treatment. Clin Geriatr Med. 2022;0. 10.1016/j.cger.2022.05.011 36210087

[70] Lundberg M, Eriksson A, Tran B, Assarsson E, Fredriksson S (2011). Homogeneous antibody-based proximity extension assays provide sensitive and specific detection of low-abundant proteins in human blood. Nucleic Acids Res.

