# Supplementary material for: Effect of immunology biomarkers associated with hip fracture and fracture risk in older adults
Source: Immun Ageing. 2023 Oct 18;20:55. doi: 10.1186/s12979-023-00379-z (PMC10583364; doi:10.1186/s12979-023-00379-z)
Supplement: Supplementary file 2 — Supplementary Material 2 [file 12979_2023_379_MOESM2_ESM.docx]

**Appendix A.1 List of biomarkers measured with Olink 48 Inflammatory Target**

| **Biomarker name (Abbreviation)** | **Values < LOD** |
| --- | --- |
| C-C motif chemokine 2 (CCL2) | 0% |
| C-C motif chemokine 3 (CCL3) | 3% |
| C-C motif chemokine 4 (CCL4) | 3% |
| C-C motif chemokine 7 (CCL7) | 0% |
| C-C motif chemokine 8 (CCL8) | 0% |
| C-C motif chemokine 13 (CCL13) | 0% |
| C-C motif chemokine 19 (CCL19) | 2% |
| C-X-C motif chemokine 9 (CXCL9) | 2% |
| C-X-C motif chemokine 10 (CXCL10) | 0% |
| C-X-C motif chemokine 11 (CXCL11) | 0% |
| Eotaxin (CCL11) | 0% |
| Fms-related tyrosine kinase 3 ligand (FLT3LG) | 0% |
| Granulocyte colony-stimulating factor (CSF3) | 0% |
| Granulocyte-macrophage colony-stimulating factor (CSF2) | 88% |
| Hepatocyte growth factor (HGF) | 0% |
| Interferon gamma (IFNG) | 0% |
| Interleukin-1 beta (IL-1β) | 78% |
| Interleukin-2 (IL-2) | 95% |
| Interleukin-4 (IL-4) | 95% |
| Interleukin-6 (IL-6) | 0% |
| Interleukin-7 (IL-7) | 0% |
| Interleukin-8 (CXCL8) | 3% |
| Interleukin-10 (IL-10) | 0% |
| Interleukin-13 (IL-13) | 90% |
| Interleukin-15 (IL-15) | 0% |
| Interleukin-17A (IL-17α) | 20% |
| Interleukin-17C (IL-17C) | 0% |
| Interleukin-17F (IL-17F) | 68% |
| Interleukin-18 (IL-18) | 0% |
| Interleukin-27 (IL-27) | 0% |
| Interleukin-33 (IL-33) | 32% |
| Interstitial collagenase (MMP1) | 18% |
| Lymphotoxin-alpha (LT-α) | 0% |
| Macrophage colony-stimulating factor 1 (CSF1) | 0% |
| Macrophage metalloelastase (MMP12) | 0% |
| Oncostatin-M (OSM) | 0% |
| Oxidized low-density lipoprotein receptor 1 (OLR1) | 0% |
| Pro-epidermal growth factor (EGF) | 2% |
| Protransforming growth factor alpha (TGF-α) | 0% |
| Stromal cell-derived factor 1 (CXCL12) | 0% |
| Tumor necrosis factor (TNF) | 0% |
| Tumor necrosis factor ligand superfamily member 10 (TNFSF10) | 0% |
| Thymic stromal lymphopoietin (TSLP) | 97% |
| Tumor necrosis factor ligand superfamily member 12 (TNFSF12) | 0% |
| Vascular endothelial growth factor A (VEGFA) | 0% |

Note: Grey shade indicates biomarkers with 35% or more of the values below the lower limit of detection (LOD).

**Appendix A.2: Non-significantly associated Olink serum biomarker levels by groups.**

| **Biomarker name (Abbreviation)** | **Non-fracture group (n=20)** | **Fracture group (n=20)** | **Difference** | **SE of difference** | **t-ratio** | **df** | **Adjusted-P value** | **Adjusted-P value (-log10)** |
| --- | --- | --- | --- | --- | --- | --- | --- | --- |
| CCL8 | 53.73 | 39.41 | 14.32 | 6.72 | 2.13 | 28 | 0.4510 | 0.3458 |
| IL-33 | 0.51 | 0.38 | 0.13 | 0.12 | 1.09 | 26 | 0.9180 | 0.0372 |
| CXCL12 | 185.50 | 202.00 | -16.57 | 14.62 | 1.13 | 24 | 0.9180 | 0.0372 |
| OLR1 | 404.80 | 400.70 | 4.17 | 83.60 | 0.05 | 28 | 0.9847 | 0.0067 |
| IL-27 | 7.10 | 35.42 | -28.32 | 7.55 | 3.75 | 27 | 0.0236 | 1.6280 |
| CXCL9 | 98.40 | 170.00 | -71.59 | 32.12 | 2.23 | 26 | 0.4111 | 0.3860 |
| TGF-α | 26.34 | 49.88 | -23.54 | 6.66 | 3.53 | 28 | 0.0369 | 1.4330 |
| TNFSF12 | 729.20 | 536.50 | 192.70 | 49.49 | 3.89 | 27 | 0.0175 | 1.7560 |
| CCL11 | 180.40 | 119.80 | 60.64 | 15.61 | 3.89 | 28 | 0.0175 | 1.7560 |
| IL-7 | 3.07 | 5.10 | -2.03 | 0.58 | 3.51 | 28 | 0.0376 | 1.4240 |
| IL-18 | 334.30 | 274.00 | 60.26 | 32.11 | 1.88 | 27 | 0.5819 | 0.2351 |
| CCL13 | 238.90 | 150.30 | 88.65 | 29.90 | 2.97 | 28 | 0.1101 | 0.9581 |
| TNFSF10 | 548.80 | 373.50 | 175.30 | 51.00 | 3.44 | 28 | 0.0435 | 1.3620 |
| CXCL10 | 106.50 | 200.90 | -94.44 | 24.94 | 3.79 | 26 | 0.0233 | 1.6320 |
| IFNG | 0.23 | 0.24 | -0.01 | 0.05 | 0.22 | 25 | 0.9847 | 0.0067 |
| IL-10 | 7.52 | 26.11 | -18.60 | 7.56 | 2.46 | 27 | 0.2831 | 0.5480 |
| CCL19 | 84.40 | 108.70 | -24.26 | 16.35 | 1.48 | 24 | 0.7708 | 0.1131 |
| TNF | 83.09 | 45.60 | 37.49 | 19.88 | 1.89 | 27 | 0.5819 | 0.2351 |
| IL-15 | 13.41 | 17.52 | -4.11 | 1.14 | 3.60 | 28 | 0.0321 | 1.4940 |
| CCL3 | 73.43 | 33.33 | 40.10 | 21.84 | 1.84 | 24 | 0.5819 | 0.2351 |
| CXCL8 | 68.92 | 57.09 | 11.83 | 20.35 | 0.58 | 24 | 0.9847 | 0.0067 |
| MMP12 | 363.50 | 523.80 | -160.40 | 49.99 | 3.21 | 23 | 0.0825 | 1.0840 |
| CSF3 | 102.50 | 269.10 | -166.60 | 61.71 | 2.70 | 26 | 0.1959 | 0.7081 |
| VEGFA | 799.30 | 939.80 | -140.40 | 129.20 | 1.09 | 28 | 0.9180 | 0.0372 |
| IL-17C | 28.05 | 65.78 | -37.73 | 11.51 | 3.28 | 26 | 0.0660 | 1.1810 |
| EGF | 567.60 | 360.40 | 207.10 | 68.11 | 3.04 | 27 | 0.0988 | 1.0050 |
| CCL2 | 592.10 | 630.30 | -38.26 | 79.39 | 0.48 | 26 | 0.9847 | 0.0067 |
| IL-17α | 0.70 | 1.78 | -1.08 | 0.51 | 2.13 | 27 | 0.4510 | 0.3458 |
| OSM | 10.03 | 21.43 | -11.40 | 3.62 | 3.15 | 26 | 0.0825 | 1.0840 |
| CCL4 | 350.70 | 311.30 | 39.36 | 94.14 | 0.42 | 25 | 0.9847 | 0.0067 |
| CXCL11 | 52.76 | 70.11 | -17.34 | 6.80 | 2.55 | 26 | 0.2525 | 0.5977 |

**Appendix A.3**: Flowchart of patients included in the study

**256**

Screening

**173** Excluded

**112** Secondary osteoporosis.

**48** Terminal illness.

**13** Declined to participate.

**43** Excluded

**24** Change intervention day.

**11** Impossible obtain sample.

**8** Died before intervention.

**83** Assessed for Eligibility

**40** Analyzed

**Appendix A.4**: Group difference (fracture vs. non-fracture) with candidate etabolite markers

|  | **Fracture**  **Group (n=20)** | | **Non-fracture group (n=20)** | | **Mean difference** | | **Ƞ^2^** | **P value^*^** |
| --- | --- | --- | --- | --- | --- | --- | --- | --- |
|  | Mean | SE | Mean | SE | Mean | SE |  |  |
| Lymphotoxin-alpha  (LT-α) | 1.68 | 0.31 | 2.85 | 0.29 | 1.16 | 0.52 | 0.191 | **0.037** |
| Fms-related tyrosine kinase 3 ligand (FLT3LG) | 121.01 | 7.34 | 76.30 | 7.34 | 44.70 | 12.89 | 0.334 | **0.002** |
| Interleukin 6  (IL-6) | 726.36 | 47.08 | 539.14 | 44.67 | 187.21 | 80.22 | 0.191 | **0.029** |
| Colony stimulating factor 1 (CSF1) | 336.15 | 82.22 | 319.67 | 97.43 | 16.47 | 156.81 | 0.001 | 0.917 |
| Chemokine (C-C motif) ligand 7 (CCL7) | 4879.64 | 562.72 | 2361.91 | 500.17 | 2517.72 | 929.60 | 0.268 | **0.014** |

* One-way analysis of covariance (ANCOVA) was performed to compare patients with fracture vs. non-fracture. All analysis were adjusted for age, sex, body mass index, and FRAX (hip and major) score. We calculated the partial eta squared (ƞ^2^) to estimate the effect size of fracture vs. non-fracture, considering the effect as small (0.0-0.13), substantial (0.13-0.26) and large (>0.26).
