# Supplementary figures and images for: Effect of immunology biomarkers associated with hip fracture and fracture risk in older adults
Source: Immun Ageing. 2023 Oct 18;20:55. doi: 10.1186/s12979-023-00379-z (PMC10583364; doi:10.1186/s12979-023-00379-z)

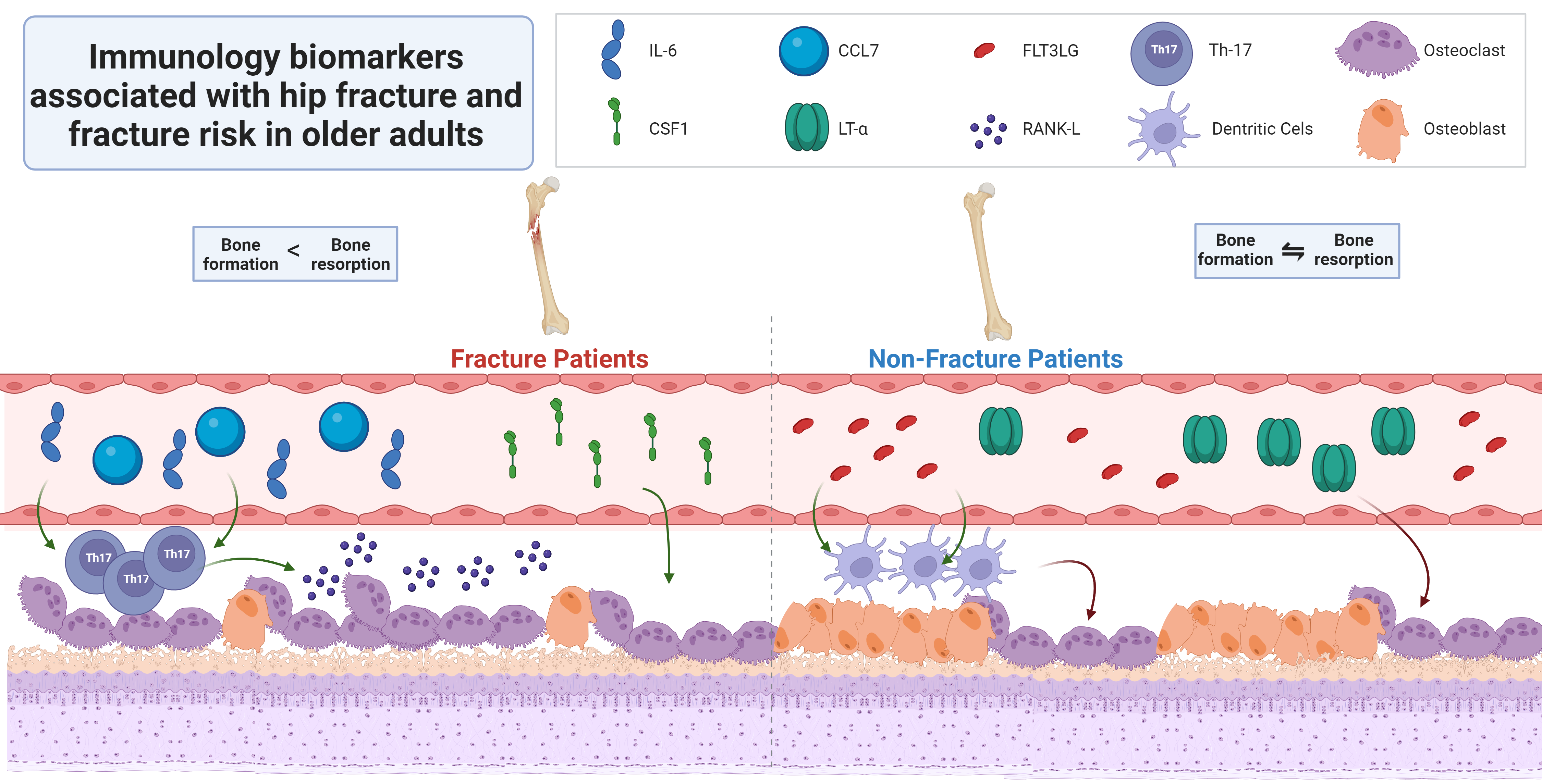

Supplement: Supplementary file 3 — Supplementary Material 3 [file 12979_2023_379_MOESM3_ESM.png]
